# Marine Invasive Algae’s Bioactive Ingredients as a Sustainable Pathway in Cosmetics: The Azores Islands as a Case Study

**DOI:** 10.3390/md22120575

**Published:** 2024-12-23

**Authors:** Marta Matos, Luísa Custódio, Catarina Pinto Reis

**Affiliations:** 1 Faculty of Pharmacy, Universidade de Lisboa, Av. Professor Gama Pinto, 1649-003 Lisboa, Portugal; martadiasmatos1313@gmail.com; 2 Centre of Marine Sciences, (CCMAR/CIMAR LA), Faculty of Sciences and Technology, University of Algarve, Campus of Gambelas, Ed. 7, 8005-139 Faro, Portugal; 3 Research Institute for Medicines, iMed.ULisboa, Faculty of Pharmacy, Universidade de Lisboa, Av. Professor Gama Pinto, 1649-003 Lisboa, Portugal; 4 Instituto de Biofísica e Engenharia Biomédica (IBEB), Faculdade de Ciências, Universidade de Lisboa, Campo Grande, 1749-016 Lisboa, Portugal

**Keywords:** cosmetics, marine invasive species, algae, case study of Azores

## Abstract

Marine invasive species pose significant ecological, economic, and social challenges, disrupting native ecosystems, outcompeting local species and altering biodiversity. The spread of these species is largely driven by global trade, shipping, and climate change, which allow non-native species to establish themselves in new environments. Current management strategies, including early detection, rapid response, and biosecurity measures, have had some success, but the complexity and scale of the problem require continuous monitoring. This review explores the possibility of using some marine invasive species as skincare ingredients and explores the Azorean islands as a case study for the valorization of biomass. Additionally, this review addresses legislative barriers that delay the development of sustainable cosmetic markets from invasive species, highlighting the regulatory landscape as a critical area. It concludes that marine invasive species present a regional and global problem that requires regional and global solutions. Such solutions strongly need to address environmental impacts and net socioeconomic benefits, but such solutions must also consider all regional differences, technical capacities and financial resources available. Thus, as a future perspective, strategies should emphasize the need for international collaboration and the development of more effective policies to prevent the spread of invasive species. There is still much work to be completed. By working together, the biodiversity for future generations will be better monitored and explored.

## 1. Introduction

Invasive species are one of the most relevant ecological concerns due to their growing negative effects, and humans present themselves as the main facilitators for the introduction of exotic species [1]. In the case of marine organisms, the continuous increase in invasive species is attributed to the continuous expansion of shipping transport, aquaculture, and aquarium trade [2]. The invaded habitats experience high levels of human disturbance, which contributes to the spread of propagules, and exotic species are rapidly dispersed [3,4]. When established, they displace native species, altering food chains, nutrient cycling and sedimentation [3,5], while also promoting a loss of genetic biodiversity and biotic homogenization across the globe [4,6,7]. Economically, they interfere with fishing and aquaculture businesses, deteriorate marinas and ports, interfere with tourism and marine recreational activities, while also representing an expense with control and eradication measures [3,5]. These effects are also associated with the production of great amounts of biomass—which is yet to be valorized and usually ends up in landfills or incineration [8]. Thus, marine invasive species have unexplored potential as biofuels [9], food, cosmetics, biofertilizers and in pharmaceutical development and biotechnology [10].

Focusing specifically on invasive algae, their introduction in exotic habitats is frequently unintentional, through ballast water, hull fouling and accompanying aquaculture species [11,12]. These accidental vectors represent more than 75% of documented algae introductions [12]. In terms of invasion processes, the *Propagule Pressure Hypothesis* is especially relevant in these introductions. Propagules are the set of individuals released in an exotic environment, and the pressure exerted by them can be influenced by the propagule size, how distant the donor region is, or if the donor region has had any environmental changes that benefit invasion processes [1]. Most studies attribute Propagule’s success to anthropogenic activity, disturbance of the habitat and resources’ availability [13]. Another important theory in explaining seaweed invasions is the *Enemy Release Hypothesis* (ERH), in which “a reduced pressure by enemies in the non-native range contributes to invasion success” [14]. The ERH is measured on population growth and is based on three arguments: (i) natural enemies are important for ecosystem balance, (ii) exotic species are not as influenced by enemy pressure as native species and (iii) alien species benefit from less enemy pressure, increasing their competitiveness and demographics [15]. Lastly, the *Novel Weapon Hypothesis* (NWH) suggests that some exotic species shift from having a limited distribution in their original habitat to spreading aggressively in a new one due to the release of novel biochemicals or soil microbes that inhibit neighboring species [16]. This phenomenon, in plants, is called allelopathy [17]. While it is not strictly meant to have a negative effect on other species, it seems that the new competitors are more sensitive to these biochemicals than the plants’ natural enemies, since they have adapted over time [16].

**Figure 1:**
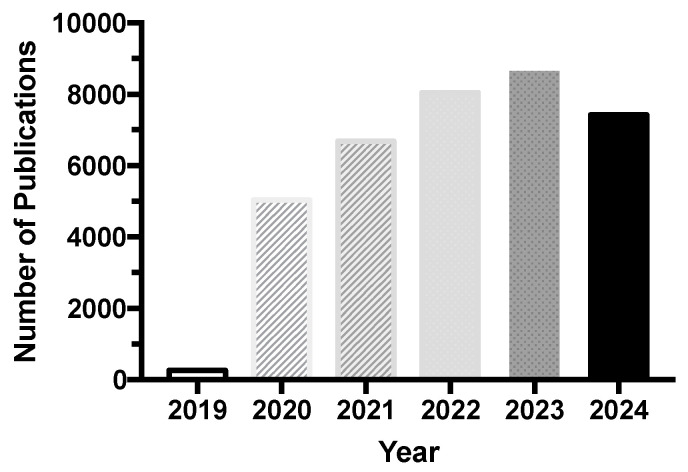
Number of publications in Google Scholar using “algae biomass cosmetics” as keywords, from 2019 to 2024 (incomplete year).

The demand for cosmetics made from natural ingredients has been steadily increasing due to claims of safety and sustainability [18,19]. This type of product offers the possibility of a circular economy and customer’s satisfaction [18]. In turn, algae-derived ingredients offer unique activities in cosmetics, with a wider variety of compounds than terrestrial plants, and allow for substantial biomass production [19,20]. Specifically, the idea of using algae biomass as a resource for cosmetic ingredients has been gaining popularity over the years. Figure 1 illustrates the number of publications available in the last five years on Google Scholar, one of the most used platforms. The keywords used in the search were “algae biomass cosmetics”, and results from the year 2024 were obtained on 5 December 2024.

This review aims to showcase the potential of using marine invasive algae as ingredients for cosmetics, as it not only represents better invasive species management but also allows for the generation of revenue sustainably. Besides detailing some of the most damaging marine invasive species worldwide, this work explores the example of the Azorean islands as a prime subject for biomass valorization and the legislative barriers for this upcycling.

## 2. Methods

This review compiles the available online literature on Google Scholar, ScienceDirect and on the PubMed database, ranging between 1990 and 2024 regarding publication date. Recently published literature (from the last 5 years) was prioritized. The used keywords included “cosmetics”, “marine”, “invasive species”, “algae” and “Azores”, alone or in combination. Legislation from various countries was also used in this review.

The invasive species detailed in this review were chosen based on the Global Invasive Species Database (GISD), widely used by policy makers and investigators, and based on peer-reviewed information. The “100 of the World’s Worst Invasive Alien Species” list was built regarding the species’ impact on biodiversity and on human activities [21]. This list was prioritized and consulted from March to October 2024. In the section about the Azorean archipelago, the marine invasive species were chosen based on Gabriel et al.’s inventory [22].

The mentioned Azorean companies that market algae were chosen by inputting the keywords “empresas”, “algas”, “Açores” and “cosméticos” (“company”, “algae”, “Azores” and “cosmetics”, respectively, in English) into the Google search engine, from January to June 2024. The authors declare no conflicts of interest nor professional affiliations with the mentioned companies.

## 3. Invasive Marine Algae

In invasive species research, terrestrial habitats are much more frequently investigated [2,23,24]. This results in a continuous increase in introduction reports of algae, insects, mollusks and crustaceans [25], with no apparent sign of eventually decreasing [11]. For example, introduction reports of the algae *Undaria pinnatifida* have been increasing gradually in the last 50 years, having hit the climax of annual reports between 2005 and 2010 [4].

**Figure 2:**
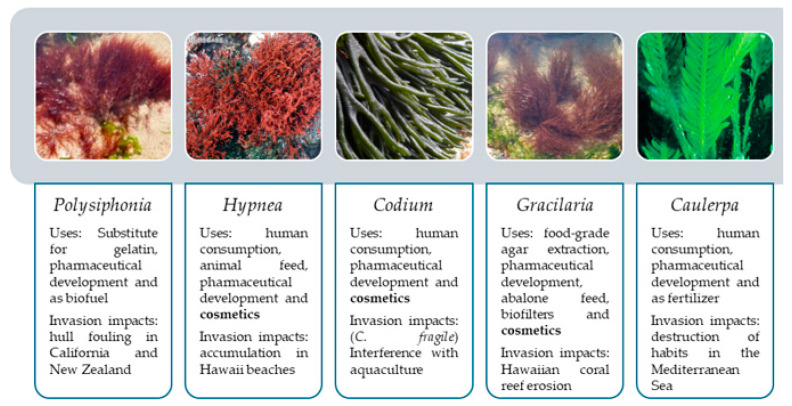
Genera with most reported invasive taxa, as reviewed by Thomsen et al. [12], and their described uses and impacts [27,28,29,30,31,32,33,34,35,36]. *Polysiphonia* image by Bárbara Ignacio, available under a Creative Commons Attribution-Noncommercial-Share Alike 4.0 License [37] at [38]. *Hypnea* image reproduced with permission from Olivier De Clerck, published by AlgaeBase [39]. *Codium* image by Manuela Lopes, published by “Casa das Ciências”, available under a Creative Commons Attribution-Noncommercial-Share Alike 4.0 License [37] at [40]. *Gracilaria* image by António Palmeira, published by OMARE, available under a Creative Commons Attribution-Noncommercial 4.0 License at [41]. *Caulerpa* image reproduced with permission from the Australian Institute of Marine Science, published by New Zealand Marine Biosecurity [42].

Thomsen et al. [12] reviewed the literature regarding non-native and cryptogenic algae, aiming to update previous compilations. The authors identified 346 taxa of introduced species, in which *Polysiphonia*, *Hypnea* and *Codium* were the top three genera with most invasive taxa [12]. Figure 2 details the described uses of the species mentioned by Thomsen et al., alluding to the possibility of upcycling this biomass.

In fact, worldwide, tons of algae are removed yearly from beaches due to their harmful consequences on tourism [9,10,26] and production of greenhouse gases [10], but usually end up in landfills [8]. Algae bio-waste has a great potential for valorization [10], and the removal of beach-cast algae brings economic development to the region, while also assuring beach aesthetics and frequentation by tourists. However, these seaweeds also play an important ecological role in beach ecosystems. They prevent predation for some macrofauna species, nourish some invertebrates and maintain ecosystem balance in beaches. Thus, the impact caused by the removal of these seaweeds should be measured when managing this resource [26]. Australia, Ireland, Canada and New Zealand are examples of countries which have been utilizing beach-cast seaweeds for various purposes. In Australia and Ireland, algae have been used for fertilizer and animal feed production [10], while in Canada, *Mazzaella japonica*, an introduced species, has been collected from beaches since 2007, representing an important source of carrageenan for the country [26].

In the following sections, various invasive algae’s ingredients are explored with the aim of being formulated into cosmetics, along with other possible uses. In the GISD “100 of the World’s Worst Invasive Alien Species” list, only *Caulerpa taxifolia* and *Undaria pinnatifida* were considered, but the authors seemed fit to also include *Sargassum muticum* due to its dire effects in the European coast [43]. It must be noted that only *C. taxifolia* is included in the genera mentioned by Thomsen et al. [12] showing that the number of reports does not translate into the severity of the species’ impact.

### 3.1. Sargassum muticum’s Bioactive Ingredients

This brown seaweed is native to the Japanese and Chinese coasts, having reached Europe and America due to oyster shipments. Currently, it endangers multiple coastal habitats due to its ability to expand in a “canopy” manner, limiting solar light access from other species located on the bottom of the ocean and decreasing water oxygenation [44,45] (Table 1). *S. muticum* can reach great dispersal by breakage of fronds, which demonstrate great buoyancy due to the species’ air bladders, by releasing planktonic propagules [45].

Chemically, *Sargassum* spp. has a great variety of components, being sulfated polysaccharides and polyphenolic compounds the most abundant metabolites, followed by meroterpenoids, sterols, meroditerpenoids and aminoacids [46,47,48,49]. This variety allows the species’ biomass to be potentially utilized in fertilizers, food additives, biodegradable materials, textiles and cosmeceuticals [50].

In 2022, Susano et al. extracted *S. muticum* fractions and tested their anti-collagenase, anti-elastase, anti-hyaluronidase and anti-tyrosinase activities. The ethyl acetate fraction of the algae was able to markedly inhibit collagenase, hyaluronidase and tyrosinase, giving the seaweed great anti-aging power. Regarding hyaluronidase, their results had surpassed past experiments with *S. polycystum* species, with IC_50_ values ranging between 20.97 to 268.66 μg/mL. Susano et al.’s experiments revealed IC_50_ values of 23.7 μg/mL and 72.3 μg/mL, towards hyaluronidase and tyrosinase, respectively [44]. Another study showed that *S. muticum* extract reduced the expression of tyrosinase-related proteins (TRP) and dramatically inhibited the microphthalmia transcription factor (MITF) [51]. Thus, it is clear that this alga is a potential ingredient for skin whitening and anti-aging cosmetics [44].

*S. muticum*’s polysaccharides, namely fucoidans, alginate and laminarin [52,53], represent about 27% of the alga’s secondary metabolites [46], and have a high potential for being used in photoprotection, while also having moisturizing properties [48,54] and enhancing the product’s sensorial experience [54]. The seaweed’s photoprotective capabilities have been proven in recent studies [44,53], despite not being one of the most studied algae in *Sargassum* spp. [44,48].

Fucoidan, with its main monomer being fucose, is one of the main sulfated polysaccharides present in *Sargassum* species [55,56]. The anti-aging properties of fucoidan are attributed to the inhibition of zinc-dependent metalloproteinases (MMPs) [53,56], collagenase and elastase [53,54,57], besides increasing the expression of type I procollagen synthesis [53,57]. Thus, fucoidan contributes to the maintenance of an intact extracellular matrix even when exposed to ultraviolet (UV) radiation, giving *S. muticum* photoprotective properties [44,56,57]. Sulfated polysaccharides from brown algae have also shown promising inhibition of tyrosinase [53,54], specially fucoidan [56,58]. Low-molecular-weight fucoidan from *S. fusiforme* has revealed its ability to reduce tyrosinase’s activity and had the same effects in a melanoma cell line [53].

Regarding the second most abundant molecules in *S. muticum*, algal polyphenols can be classified into flavonoids and non-flavonoids [58], making up to 20% of the algal extract [46]. In *S. muticum*, notable non-flavonoid phenolic compounds include phenolic acids and phlorotannins, both having antioxidant, anti-inflammatory, anti-tumoral, and anti-aging activities [49,59]. They act not only as radical scavengers [60], but also can downregulate MMP activation and suppress inflammation by acting upon ciclooxigenase-2 (COX-2) [61]. Phlorotannins seem to have the ability to absorb UV radiation, acting as a radiation filter [53], and, along with flavonoids, show great activity in tyrosinase inhibition for skin whitening purposes [61]. Yu et al., in 2019, extracted a polyphenol fraction from *S. muticum* using 38% ethanol. After further extractions using various solvents, the authors demonstrated excellent radical scavenging in the ethyl acetate phase, reducing the reactive oxygen species (ROS) level in human fibroblasts previously irradiated by UV radiation [49].

In *Sargassum* spp., the most abundant polyphenolic compounds are fucols, phlorethols, fucophlorethols, fuhalols and eckols [62]. In a 2024 study, Zheng and collaborators extracted phlorotannins from powdered *S. muticum* using ethanol, earning a yield of 24.90 ± 0.52 mg of phloroglucinol equivalent per gram of polar parts. Afterwards, they characterized the obtained phlorotannins by ion mass spectra, identifying them as dibenzodioxine-1,3,6,8-tetraol, tetraphlorethol, pentaphlorethol, and fucopentaphlorethol, all from the phlorethol group [63]. Phloretols seem to be able to upregulate nuclear factor erythroid 2-like (Nrf2) regulation via the extracellular signal-regulated kinase (ERK) phosphorylation [64], besides being excellent antioxidants, such as other phlorotannins [53].

*S. muticum* exhibits a diverse and abundant profile of phenolic acids and flavonoids [52]. In 2023, Jesus et al. extracted phenolic compounds from this specie using eutectic solvents, and quantified them through high-performance liquid chromatography. The solvent with best yield was proline combined with propylene glycol, with values of 15.26 ± 0.08 mg of gallic acid equivalents per g of dry weight, and the phenolic acids identified in biggest amount were salicylic acid, 3,4-dihydroxybenzoic acid and gallic acid. In terms of flavonoids, catechin was the most abundant in these experiments [59].

Meroditerpenoids, accounting for about 8% of the algal secondary metabolites [46], result from biosynthesis pathways that have terpenoids as a substrate, exhibiting great chemical variability [65]. Compounds like sargachromenol, sargachromanol D, sargaquinoic acid and sargahydroquinoic acid are considered meroterpenoids [66], and, curiously, are mostly obtained from marine organisms or fungi [65], being the brown algae genus *Cystoseira* described as seaweed with high meroditerpenoid content [67]. Terpenoid compounds have been shown to modulate the nuclear factor kappa B (NF-kB) signaling pathway, making them powerful anti-inflammatory ingredients [66], having exhibited antioxidant activity and potential tyrosinase’s inhibitors [57].

In 2015, Balboa et al. isolated tetraprenyltoluquinol chromane meroterpenoid (TPM) from powdered *S. muticum* and evaluated its effects on human fibroblasts. They concluded that TPM minimized the effects of photodamage, showing high cell viability when compared to only UV-irradiated cells, and inhibited the accumulation of intracellular ROS [68].

Brown algae’s main pigments are composed of chlorophyll a and c, fucoxanthin and other carotenoids, and have reportedly shown abilities of UV ray absorption [53]. Fucoxanthin is the main carotenoid in brown algae [57] and has shown antioxidant properties by radical scavenging [56], decreasing intracellular ROS in mice and inhibition of MMPs [53]. It can also inhibit tyrosinase activity in UVB-irradiated mice, playing a role in hyperpigmentation [53,56]. In macrophages, fucoxanthin from *S. muticum* was able to decrease nitric oxide production stimulated by lipopolysaccharides, also inhibiting the production of pro-inflammatory molecules [69], showing great anti-inflammatory potential [44,52]. Therefore, fucoxanthins reveal themselves an attractive ingredient for anti-aging skincare and to combat inflammation [61,70], reducing wrinkle formation and inhibiting epidermal hypertrophy [71].

### 3.2. Caulerpa taxifolia’s Bioactive Ingredients

*C. taxifolia* is a green alga found in warm, tropical waters, such as the Indian Ocean, that was accidently introduced in the Mediterranean Sea, and that has spread over hectares of seabed [72]. The seaweed can grow over rock, gravel or sand, over-competing existing seagrasses and stopping invertebrates from grazing due to the release of toxins [73], and is widely used in human consumption, water purification in the aquaculture industry, and as a fertilizer (Table 1) [74].

The *Caulerpa* genus is still chemically understudied, despite its identified antimicrobial, cytotoxic, immunostimulatory, hepato- and cardioprotective and hypolipidemic properties [36]. In various green algae, natural pigments, polyunsaturated fatty acids (PUFAs), lipids, proteins and polysaccharides have been identified [75], while *C. taxifolia* has proteins, caulerpenyne derivatives, and sulfated polysaccharides as frequently identified compounds [36].

*Caulerpa* spp. have in their composition sulfated heteropolysaccharides, built essentially with galactose, mannose and xylose. Just like in the previously mentioned algae, it is thought that these sugars have anti-inflammatory and antioxidant activities [74,76]. In a 2021 study, Bayro et al. extracted a crude polysaccharide fraction from *C. taxifolia*, with a yield of 0.81%. The authors studied the algae’s radical scavenging by the DPPH method, concluding that the results were unsatisfactory when comparing to other studies performed on polysaccharides. Those authors attributed these results to the fraction’s low sulfate content, which they quantified, and that can compromise the antioxidant activity [77]. In a separate study conducted by Lee et al., a polysaccharide-enriched extract derived from *C. microphysa* demonstrated significant anti-allergic and anti-inflammatory properties, along with relevant moisture retention for skin applications [78].

*C. taxifolia*’s phenolic components comprise tannins and flavonoids [75], which have been previously mentioned to have marked antioxidant activity. Reportedly, the flavonoids found in *Caulerpa* spp. are quercetin and apigenin [58]. Quercetin has been shown to inhibit MMP-1 expression in cells exposed to UV radiation, while also downregulating activator protein-1 (AP-1) and NF-κB pathways, thereby preventing collagen degradation. Additionally, it reduces COX-2 expression, establishing its role as an effective molecule in combating skin aging and inflammation [79]. Regarding apigenin, it hinders calcium ion influx in fibroblasts, which is essential for mitogen-activated protein kinase (MAPK) function and, consequently, MMPs collagen degradation caused by UV ray exposure. Moreover, this molecule is potentially able to act on atopic dermatitis, psoriasis and overall pruritus [80].

In 2018, Wiraguna et al. investigated the effects of various Indonesian *Caulerpa* sp. as a topical polyphenolic extract on mice skin and found that MMP-1 levels significantly decreased in mice in which had been applied the seaweed. They also corroborated that the vitamin content of the algae, comprising vitamin A, C and E, downregulated MMP-1 expression [81].

The primary alkaloids isolated from green algae of the *Caulerpa* genus include caulerpin, racemosins, caulersin and caulerpenyne [82,83], which have a unique chemical structure and interesting pharmacological properties [36]. The sesquiterpene caulerpenye, considered the major secondary metabolite from *C. taxifolia*, has shown cytotoxic activity against multiple cells lines, proving its anti-tumoral activity [84]. Other sesquiterpenes, cauterpals A and B, were identified in *C. taxifolia*, and seemed to have an inhibitory activity on human protein tyrosinase phosphatase [75], which acts upon diseases like diabetes and obesity [85]. On the other hand, caulerpin isolated from several *Caulerpa* species has shown antinociceptive, anti-inflammatory [74,75,82,83], antibacterial and antiviral activities [82].

### 3.3. Undaria pinnatifida’s Bioactive Ingredients

*Undaria pinnatifida*, a species of brown seaweed, originates from the cold seas of China, Japan and Korea. It has been introduced in other regions around the globe, including the European Atlantic, French Mediterranean, Australia, and New Zealand [86]. *U. pinnatifida* thrives in various light, temperature, and salinity conditions. It exhibits remarkable fertility with a rapid growth rate and high reproductive output, releasing spores throughout the year (Table 1) [86].

In the skincare realm, it is known to have a detoxifying effect and to protect the skin from pollution, tobacco and UVA rays. Additionally, *U. pinnatifida* is known for its nourishing, revitalizing, and antioxidant properties, contributing to overall skin health [70].

*U. pinnatifida*, just like other brown algae, is especially rich in fucoidan. In this case, it is typically extracted from its sporophyll, which is where the algae’s sporangia are formed. This algae’s polysaccharides have good moisture retention properties in skin [56], and its fucoidan exhibits significant primary and secondary antioxidant activities, which involve breaking chains of free radicals and scavenging them to produce more stable, non-radical products [54]. Furthermore, a 2015 study by Fitton et al. demonstrated the efficacy of *U. pinnatifida* extracts, which contained 85% fucoidan, in boosting the expression of sirtuin 1 (SIRT1), a protein associated with longevity and anti-aging effects. The authors also tested the extract’s ability to protect the skin from UV radiation, reduce wrinkle depth, and modulate skin immunity, providing soothing and protective results [87].

Additionally, the wound-healing properties of a topical low-molecular fucoidan extract from *U. pinnatifida* showed superior effects on wound contraction and faster closure times compared to a commercial product containing *Centella asiatica*, due to its anti-inflammatory properties. In molecular terms, these results are most likely due to fucoidans being able to reduce neutrophil adhesion and leukocyte recruitment, while also inhibiting the production of pro-inflammatory cytokines [61].

The anti-photoaging properties of *U. pinnatifida* fucoidan-rich extracts have been further supported by their inhibitory activity against tyrosinase, the enzyme responsible for hyperpigmentation. The algae’s extract has also been found to inhibit melanogenesis by targeting the α-melanocyte-stimulating hormone (α-MSH), offering potential benefits for reducing age spots and increasing skin brightness [88].

Finally, the alginate content of *U. pinnatifida* is also worth mentioning. A 2021 study by Queffelec et al. aimed to extract bioactive fractions of the algae, obtaining the highest yield of 4.5% after a hydrothermal treatment at 160 °C. This extracted alginate presented good rheological properties that could be an alternative in cosmetic usage [89] but also with other clinical uses [90,91,92].

As discussed in *S. muticum*, brown seaweeds have a high content of phenol compounds [62]. In a study by Shen et al., polyphenolic compounds from various algae were extracted, including *U. pinnatifida*, in which phloroglucinol pentamer and caffeic acid were identified in the largest amounts, followed by trifuhalol and phloroglucinol trimer [93]. Caffeic acid, a phenolic acid, is well-known for its antioxidant and antimicrobial properties [94], while phloroglucinol, the monomeric unit of phlorotannins, shares similar bioactive properties [62]. Ferreira et al. created a wound dressing made with gelatin and chitosan and infused them with phlorotannin extract from *U. pinnatifida*. Not only did the mesh inhibit *Pseudomonas aeruginosa* and *Staphylococcus aureus* growth, which are bacteria responsible for wound infections, but it also did not show cytotoxic effects on dermal fibroblasts. Additionally, the phlorotannin extract also exhibited antioxidant activity, which is crucial in combating ROS produced by macrophages during wound healing [95]. In other experiments, Ferreira et al. extracted phlorotannins from *U. pinnatifida*, testing them for antioxidant and anti-inflammatory bioactivity. The antioxidant activity was in line of other brown seaweed extracts, while anti-inflammatory properties were proven with a 47% decrease in nitric oxide in mouse macrophages [96].

While marine microalgae are currently being extensively studied for their lipid content, macroalgae also contain bioactive compounds such as polyunsaturated fatty acids (PUFAs), tocopherols and sterols [97]. Recently, Stabili et al. analyzed the lipidic extract of *U. pinnatifida*, identifying phytosterols, triacylglycerols (TAGs), phospholipids, as well as both saturated and unsaturated fatty acids in the seaweed. The main sterol identified in the algae was fucosterol [98]. This molecule has shown antioxidant, antiproliferative, anti-photodamage, and anti-inflammatory effects in cells [57].

PUFAs are formed by two fatty acid series, a ω-3 series and a ω-6 series, and their ratio alters the molecule’s bioactivity [99]. This molecule’s high content in seaweed gives them antioxidant properties and helps with the skin barrier’s management [57,70], which shows the anti-aging potential of these ingredients. In fact, seaweeds present a very interesting source of PUFAs, since their content is higher than in plants [53]. The high PUFA content in *U. pinnatifida* has demonstrated significant anti-inflammatory properties, as evidenced in studies on mouse ear edema and erythema [100,101]. Commonly found PUFAs in algae are γ-linolenic, arachidonic, eicosapentanoic and docosahexanoic acids [57]. They play a crucial role in skin health, acting as emollients, preventing skin water loss and activation of MMPs [61], and having anti-allergic and anti-inflammatory activities [53]. Eicosapentaenoic acid (EPA) and docosahexaenoic acid (DHA) have been shown to modulate the inflammatory process by indirectly inhibiting interleukin-1β (IL-1β) and tumor necrosis factor-alpha (TNF-α) production via the COX-2 pathway [100]. Furthermore, *U. pinnatifida* has been identified as an excellent source of EPA [102]. *U. pinnatifida* is, primarily, the most utilized alga species for the extraction of fucoxanthin, due to its remarkable high content, which can reach up to 10% in the lipid fraction [71].

Matsui et al. used a non-edible portion of *U. pinnatifida*, referred to as the “mikine part” and typically discarded as industrial waste during seaweed farming as a source of fucoxanthin. This fucoxanthin extract showed promising results in relation to filaggrin, a crucial molecule that is downregulated by exposure to UV radiation and assures the management of the skin barrier. Fucoxanthin extracted from *U. pinnatifida* has been shown to provide UV protection and prevent sunburn, highlighting its potential as a valuable natural ingredient for skincare applications [103].

A 2022 study by Lourenço-Lopes et al. identified and quantified pigments in nine brown algae species, including *U. pinnatifida*, which yielded 10.5 mg of pigments per gram of algal dry weight. The researchers identified fucoxanthin, chlorophyll-a, and β-carotene, with *U. pinnatifida* and *Laminaria saccharina* standing out as the richest sources of these pigments [104]. These findings highlight the potential of *U. pinnatifida* for inclusion in cosmetic formulations [53,56].

**Table 1:** Summary of *Sargassum muticum*, *Caulerpa taxifolia* and *Undaria pinnatifida*’s impacts and their possible uses.

Species	Impact	Remarkable Ingredients	Other Described Uses
*S. muticum*	Habitat destruction, losses in fishing and tourism [105].	Polysaccharides and polyphenols Anti-aging Antioxidant Skin brightening	Fertilizers Food additives Biodegradable materials Textiles Pharmaceutical development
*C. taxifolia*	Habitat destruction and losses in fishing [106].	Polyphenols and alkaloids Anti-aging Antioxidant Anti-inflammatory	Human consumption Pharmaceutical development Water filter Fertilizer Decorative plant
*U. pinnatifida*	Biofouling in aquaculture [107]	Fucoidans and fucoxanthin Antioxidant Skin brightening UV protection	Human consumption Pharmaceutical development

## 4. Case-Study: Marine-Derived Ingredients and the Azores Islands

### 4.1. The Azores Archipelago as a Susceptible Ecosystem for Thriving Invasive Species

In the northeast Atlantic Ocean are located the nine Azores islands, which belong to the Macaronesia region [108]. These oceanic islands are organized in three groups: the western group, to which belong Corvo and Flores islands; the central group, including Faial, Pico, Graciosa, São Jorge and Terceira islands; and the eastern group, only comprising São Miguel and Santa Maria islands [109].

The archipelago’s climate is influenced by the Azores Anticyclone, whose position and intensity impact the weather conditions experienced in the islands. Thus, the Azores islands are characterized by high levels of humidity, mild temperatures, low rates of sunshine, regular and abundant rainfall and vigorous winds [110].

**Figure 3:**
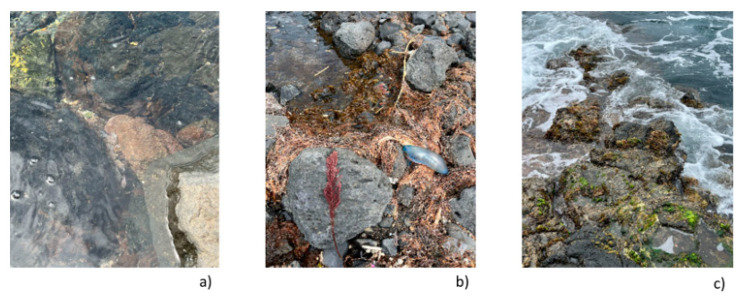
Typical species found in the Azorean intertidal area. Captured on 21 April 2024 at (**a**) Atalhada, (**b**) Cerco da Caloura and (**c**) Porto da Caloura, all in São Miguel, Azores.

The Azores lie on the border between temperate waters to the north and subtropical waters to the south. The oceanic domain is characterized by the Azores Current, fed by the Gulf Stream [111], and it is home to various cetaceans, fish, and, in coastal areas, starfish, limpets and barnacles [112] (Figure 3a–c). However, the Azores islands, upon discovery in the 15th century, had a very different landscape. The archipelago was covered by Laurel Forest, possibly comprising most of the vegetation. Today, the original insular forest ecosystem is restricted to about 5% of the total area of Azores, and about three quarters of existing species are exotic. This highlights how human disturbance has changed the islands’ terrestrial ecosystem, and, undoubtedly, the same has happened to the Azorean marine habitats. In fact, the biggest Azorean urbanizations are in coastal areas, having led to the degradation and near extinction of natural habitats [109]. This phenomenon, however, is not exclusive to the Azores. Like other oceanic islands, the Azorean islands are fragile ecosystems, with particular biodiversity and intense endemism [108]. Islands make up only 5.3% of the global land area and are, at the same time, notable hotspots of biodiversity and epicenters of biodiversity loss. Insular habitats have undergone 61% of known extinctions and currently hold 37% of critically endangered species [113]. Table 2 describes probable explanations for islands to be susceptible to invasions, along with their transposition to Azorean context. Thus, it is clear that the Azorean archipelago is a textbook case of the problems also verified in islands like Hawaii [114], and measures should be taken to prevent further loss.

While environmental and economic losses have been previously presented, tourism is an upcoming driver of the Azorean economy [109]. In February of 2024, the Azorean Statistics Regional Service published the 2023 report for tourism in the archipelago, having broken the record for hotels’ activity. There were 1.2 million hotel guests over the year, translating into a 14.8% increase in comparison to the previous year. This translates into a total revenue of EUR 157.8 million just in hotels’ stays, representing a 23.1% increase over 2022 [115]. Considering that tourists come to Azores looking for natural landscapes and contact with nature [109], invasive species’ takeover could potentially take away one of the Azores’ most profitable sectors.

In addition to regulatory considerations, the socio-economic context of the archipelago also plays a significant role in addressing the issue. Borges et al. attribute this lack of action against invasive species to insufficient awareness of the actual threats they pose, as many people prefer to protect all animals and plants, regardless of whether they are invasive [109]. In the case of marine invasive species, an overall lack of ocean literacy might be a factor. Costa et al. assessed the perspectives of school teachers in the Azores regarding the importance of teaching ocean’s preservation and its sustainability. Although teachers acknowledge the significance of this topic, more than half (55%) do not incorporate ocean’s literacy into school activities, despite living in close proximity to the sea [116].

### 4.2. Bioactive Ingredients from Invasive Algae in the Azores

**Figure 4:**
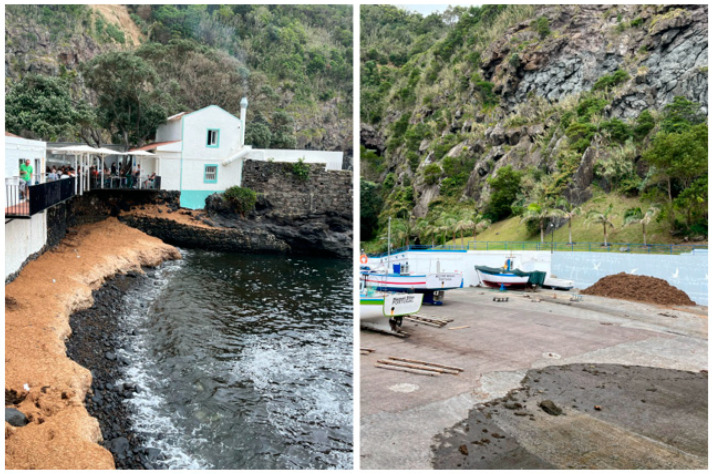
Accumulation of dried algae in the Caloura bathing site and in the Caloura port. Captured on 21 April 2024.

The introduction of exotic algae species in the Azores islands has been increasing over the last decade [117]. While a 2005 review by Cardigos et al. describes a relatively low number of introduced species [118], a more recent 2023 review by Gabriel et al. reports 42 non-native species in the archipelago. Despite this, only one invasive species, *Rugulopteryx okamurae*, has its own governmental control measures [22], despite the problems the accumulation of algae has been presenting to the island (Figure 4), namely fewer fish captures and biodiversity displacement [119]. This section aims to explore some bioactive ingredients derived from invasive algae existent in the Azorean marine environment, to evaluate the algae’s potential in upcycling and valorization. The species were chosen based on Gabriel et al.’s inventory [22].

#### 4.2.1. *Rugulopteryx okamurae*’s Bioactive Ingredients

**Figure 5:**
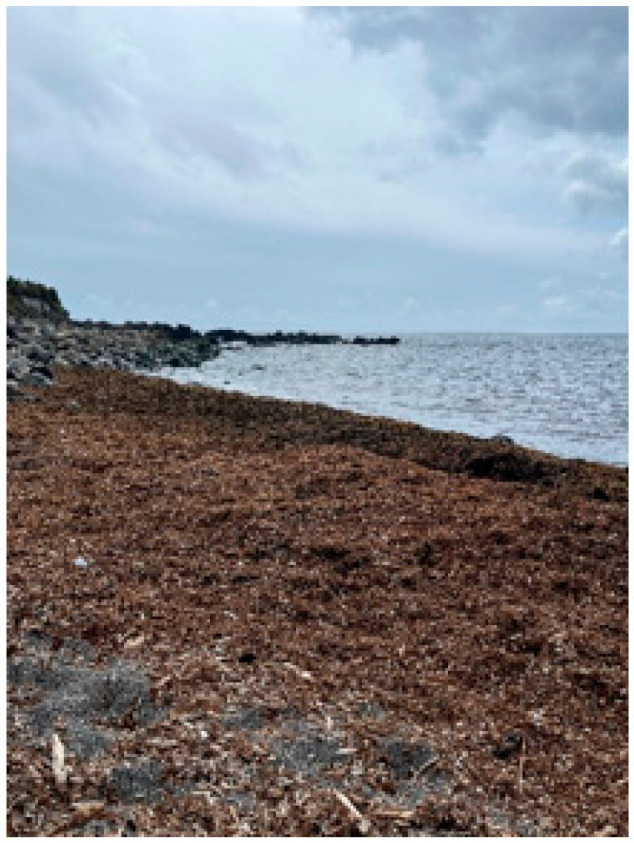
*Rugulopteryx okamurae* accumulation at Prainha de Água d’Alto. Captured on 21 April 2024.

*Rugulopteryx okamurae* is a brown alga possibly introduced to the Azores via ballast waters or ship hulls from the introduced algae community in the Strait of Gibraltar [120]. Originating from the Pacific Ocean, specifically regions like the Philippines, China, Korea, and Japan, this alga spread along the south coast of São Miguel within a year, becoming the most abundant species in some areas and covering nearly 100% of the rocky seabed [121] (Figure 5). This seaweed thrives in rocky substrates up to 30 m deep, being most common within the first 15 m. It inhabits both well-lit and dark zones and can be present year-round through dormant rhizoids. *R. okamurae* also has an highly competitive ability, producing allelopathic substances that inhibit the growth of its direct competitors for space and that deter herbivore grazing [121]. This species has significantly impacted the communities it has invaded, quickly making its way onto the European list of invasive non-native species [22], which is evident by being the only macroalga with specific legislation for its monitoring and control in the Azores [119].

To date, there are reports on possible applications of *R. okamurae*, mostly exploring its use as biomass for composting, development of bioplastics, biofertilizer and biogas produced by anaerobic digestion. This process uses microorganisms to produce biogas mainly made up of methane and depends on the biomass’ carbon/nitrogen ratio. *R. okamurae*, for optimal methane production, must be co-digested with other types of waste. Regarding composting, due to the algae’s high concentration in terpenes, its decay is arduous, and, furthermore, sesquiterpenes present toxicity to species commonly used in vermicomposting. This can be solved with the addition of food or vegetable residues. In bioplastics, this species serves as a promising source of raw material; however, its mechanical stability may be compromised due to its water absorption capacity [120]. As of 19 June 2024, this species does not appear in the European CosIng cosmetics database, indicating that its use in cosmetics marketed within the European Union has not yet been reported.

Regarding chemical composition, Vega et al. showed that the algae’s main components are carbonated compounds (total carbon or lipids) through spectrophotometric methods. Additionally, the phenolic compound content found in *R. okamurae* was similar to other brown seaweeds, but it did not reach the values observed in *Sargassum* sp. or *Cystoseira* sp. Vega et al. also tested the algae’s antioxidant and anti-acne activity, and *R. okamurae* did not exhibit a particularly high antioxidant capacity when comparing to other brown algae. However, it had good inhibitory activity towards bacteria related to skin infection, namely *Staphylococcus aureus* and *Cutibacterium acnes* [122].

As previously mentioned, common polysaccharides in brown algae are composed of fucoidans, alginate and laminarin. Santana et al. extracted alginate from *R. okomurae*, achieving a yield of approximately 74%. The resulting gel exhibited higher viscosity compared to commercial alginates, attributed to a lower mannuronic acid to glucuronic acid (M/G) ratio. Notably, it also demonstrated superior moisture retention properties relative to commercial counterparts [123], making it particularly advantageous for use in cosmetic formulations aimed at enhancing skin hydration. Another experiment by Cebrián-Lloret et al. confirmed the high percentage of alginate in this seaweed, making up 32% of the overall carbohydrate composition, and obtained a lower M/G ratio [124].

Polyphenols constitute approximately 4.5% of *R. okamurae*’s dry weight, according to experiments conducted by Cebrián-Lloret et al. This represents a relatively high content compared to other species in the Dictyotaceae family, contributing to its strong antioxidant properties [124]. In the previously mentioned study by Vega et al., phenolic compounds were identified in both *Asparagopsis armata* and *R. okamurae*. Among these, kojic acid, a potent tyrosinase inhibitor commonly used in skin-whitening formulations, was notable. Additionally, ferulic acid, recognized for its ability to inhibit free radical formation and matrix metalloproteinases (MMPs), was also identified. Ferulic acid provides protection against UV-induced skin aging [122,125].

Terpenoids are the primary molecules studied in *R. okamurae*, known for their antifungal, antibiotic, anti-inflammatory, insecticidal, and antiviral applications across various industries [122]. While these compounds had already been identified in Japan, they were found to be significantly more abundant in specimens from the Strait of Gibraltar. For instance, the concentration of dilkamural in native Japanese species was 1.9% (*w*/*w*), less than half the average concentration observed in specimens from the Strait of Gibraltar [120].

Cuevas et al. successfully isolated novel diterpenoids from *R. okamurae*, including rugukadiol A, rugukamurals A, B, and C, as well as ruguloptones A, B, C, D, E, and F. Additionally, known compounds such as dilkamurals 11 and 12, which are the primary metabolites of the extract, were also obtained. The researchers demonstrated the anti-inflammatory properties of rugukadiol A, ruguloptone A, and ruguloptone F, as these compounds were able to nearly neutralize the effects of lipopolysaccharide (LPS) stimulation on cells [126].

Another species from the genus *Dictyota*, *D. coriacea*, has reported anti-melanogenesis activity, contributing to the prevention of skin hyperpigmentation, and it is likely attributed to the diterpene components of the algae [120], which could be something worth investigating in *R. okamurae.* Within the same order, *R. okamurae* seems to exhibit a higher lipid content than species such as *Dictyopteris australis*, *Dictyota bartayresiana*, or *Stypopodium zonale*. Therefore, the alga is a great candidate for lipid extraction and inclusion in dietary supplements or cosmetics. In the study by Cebrián-Lloret et al., the fatty acid composition of *R. okamurae* was also analyzed. The findings indicate that saturated fatty acids dominate the lipid profile of the seaweed, followed by PUFA and monounsaturated fatty acids (MUFA). The most abundant fatty acids in *R. okamurae* were palmitic and myristic acids, comprising 32% and 15% of the total fatty acid content, respectively [124]. Myristic acid, in combination with palmitoleic acid and lauric acid, showed activity against skin bacteria, *Staphylococcus aureus* and *Staphylococcus epidermidis* [127], and thus the extraction of this fatty acid from *R. okamurae* could be explored in anti-acne cosmetics.

#### 4.2.2. *Asparagopsis armata*’s Bioactive Ingredients

*Asparagopsis armata*, originally from Australia, has now invaded extensive areas in the North Atlantic, Senegalese coast and the Mediterranean basin. This seaweed’s toxic metabolites inhibit competing algae fixation, reducing species richness in habitats, and have already impacted the Azorean economy by negatively affecting fishing and aquaculture [122]. In the Azores, it is the only algae that is recorded on all islands [22], due to its long history of invasion in the archipelago [118].

However, its potential is also recognized, as this alga is cultivated commercially in northern Europe for bioactive molecules extraction, such as sulphated polysaccharides with iodine and bromine groups, which are used as natural preservatives in cosmetics and anti-acne products [128]. In fact, an *A.* extract is already commercialized under the name ASPAR’AGE™. Rich in amino acids, the ingredient promises to promote skin elasticity, reduce wrinkles, and stimulate collagen production [101]. Additionally, an extract combination from the algae *Ascophyllum nodosum* and *A. armata* has reportedly offered relief to sensitive skin types. This extract alleviates tingling sensations, improves resiliency, and enhances comfort in individuals with sensitive skin [97]. In fact, Revea is an American brand that features *A. armata*’s extract in their soothing serum. The product’s anti-redness properties are attributed to the algal extract [129].

Pinto et al. evaluated the bioactivity of *A. armata*, reporting results from an antioxidant DPPH assay, which showed a radical scavenging activity of 23.6%, and a 43.2% inhibition towards tyrosinase with an extract concentration of 250 µg/mL [128]. In a separate study by Lee et al., the antioxidant, anti-tyrosinase, anti-elastase, and anti-acne properties of *A. armata* extracts were investigated. Two types of extracts were prepared: one using hydrothermal extraction (HAE) and the other using supercritical carbon dioxide extraction (SAE). The HAE extract demonstrated higher radical scavenging activity in the DPPH assay, achieving 41% at 5 mg/mL. In tyrosinase inhibition, the SAE extract was more effective, reducing tyrosinase activity by 39% at 333 mg/mL of extract [130]. These findings suggest that *A. armata* possesses anti-hyperpigmentation and anti-aging properties.

Agars and carrageenans are the most abundant structural polysaccharides in red seaweeds. Félix et al. report that, in *A. armata*, non-starch polysaccharides, in which are included agars and carrageenans, make up 71% of dry algal biomass, which is a high value for red seaweeds [131]. Agar, which includes agarose and agaropectin, produces agaro-oligosaccharides that act as antioxidants and have anti-inflammatory properties [100]. It can be useful for in vitro models to assess the efficacy of formulations [92,132]. Carrageenans are sulfated polysaccharides that, in turn, can form thermoreversible gels, which can be valuable in cosmetics as emulsion stabilizers, film formers, and hair conditioning agents [100]. In skincare, they also help moisturize the skin, enhancing its suppleness and preventing aging [133].

Red seaweeds, like *A. armata*, are rich in protein, with content ranging from 10 to 47% of dry weight, comparable to high-protein legumes like soybeans, where proteins constitute 35% of the dry mass. Protein extracts from *A. armata* have been shown to enhance skin softness, brightness, elasticity, and have anti-aging qualities, making them suitable for skincare products like creams, oils, soaps, masks, and lotions [133]. Some abundant amino acids in *A. armata* are glutamic acid, aspartic acid and leucine, representing 1.82%, 1.19% and 0.93%, respectively, of algal dry weight [131].

Mycosporine-like amino acids (MAA) are algal metabolites synthesized for solar radiation protection purposes, consisting of cyclohexenone or cyclohexenimine chromophores with glycine or iminoalcohol. So, it is expectable that they exhibit antioxidant and photoprotective properties [57]. Indeed, studies have detected MAAs in different red macroalgae, including *A*. *armata*, and highlighted their role as UV protectors and cell proliferation activators in cosmetics [56]. For example, a specimen of *A. armata* was cultivated on wastewaters from a fish farm and reported an average production of 1.75 mg of MAAs per gram of dry seaweed [134].

The primary photosynthetic pigments in red algae include chlorophyll a, carotenoids (such as lutein, zeaxanthin, and β-carotene), and phycobilins (phycocyanin and phycoerythrin), which are responsible for their distinctive coloration [53,97]. Among these, phycocyanin exhibits notable anti-inflammatory, antioxidant, and wound-healing properties [97]. In experiments conducted by Martins et al. [135] R-phycoerythrin, phycocyanin, and allophycocyanin were successfully extracted from *Gracilaria gracilis*. The use of cholinium chloride as an extraction solution significantly increased the pigment yield to 46.5%, highlighting its potential as a rich source of these phycobilins. Additionally, these pigments were evaluated for their antioxidant and radical scavenging activities, with the best results observed when the algae were harvested during winter [135].

In red algae, just like in brown seaweed, C20 fatty acids like arachidonic and eicosapentaenoic acids are predominant and show great anti-inflammatory properties [100]. Other existing fatty acids in red algae, such as palmitic acid and its derivative ascorbyl palmitate, are already used in cosmetics as emulsifiers and as anti-aging ingredients [133]. In *A. armata* and through transmethylation with fatty acid methyl esters, Rocha et al. report a total fatty acid content of 60.23 mg/g of algal dry weight. Saturated fatty acids were the most abundant type at 27.10 mg/g of dry weight, and PUFAs only resulted in 2.21 mg/g of dry weight [136].

Another common ingredient in the *Asparagopsis* genus are sterols, *A. armata* reportedly containing 0.15 to 0.32 mg/g dry weight of sterol content. These compounds exhibit excellent antioxidant properties, being remarkable radical scavengers and protecting skin from extrinsic aging. Furthermore, they also exhibit anti-inflammatory properties, potentially being useful ingredients in skin conditions like dermatitis and acne, all indicating *A. armata* as a suitable ingredient for anti-aging and moisturizing skincare formulations [137].

### 4.3. Valorization of Invasive Algae in the Azores—Is It Worth It?

An analysis of the bioactive ingredients in Azorean invasive algae highlights their potential for utilization, both in cosmetics and other applications, offering various benefits. Beyond their economic value, this exploitation could also address issues related to biomass accumulation, such as reduced fishery yields, diminished beach aesthetics caused by odor and algal build-up in water, and other associated challenges [119]. While oceanic islands like the Azores are fragile ecosystems and more vulnerable to severe invasions, there are reports of more successful invasion management on islands compared to continents [7]. This success is attributed to factors such as easier control of invasive species, more effective relocation of endemic species, impactful educational programs, and stronger policy enforcement [113]. In 2022, Spatz et al. reviewed 100 years of invasive species eradication efforts on islands, analyzing 1550 reports dating back to 1872. Their findings revealed that 1081 out of 1227 eradication attempts were successful, representing an impressive 88% success rate. Although the majority of these efforts targeted mammals (97.2%), the study underscores the effectiveness of invasive species control on islands [138]. Finally, it should be noted that the monetization of algae’s bioactive ingredients is not unexplored in the Azores. SeaExpert^®^ is a licensed Faial based algae supplier, providing species like *A. armata*, *A. taxiformis*, *Cystoseira humilis*, and others, in lyophilized and dried format [139]. In the cosmetic realm, Ignae^®^ [140] and BAMandBOO^®^ [141] are Azorean brands that have formulated their products with algae extracts and shown their value in skincare applications. As dietary supplements, Algicel^®^ uses an extract from a microalgae found in São Miguel’s freshwater lakes to produce an antioxidant supplement [142]. These companies show that Azorean entrepreneurs are aware of algae’s potential to formulate natural, sustainable products that encapsulate the Azores’ essence.

## 5. Cosmetics Derived from Invasive Species—A Brief Overview of Regulatory Framework

Using invasive species as bioingredients in cosmetics offers a unique opportunity to address environmental issues while creating innovative products. However, it requires careful navigation of regulatory frameworks to ensure safety, efficacy and sustainability. Even though the use of marine-derived ingredients is increasingly more frequent in the cosmetic industry, there is a lack of policy material regulating products produced from marine biomass.

General cosmetic regulations are also not uniform in all countries. Despite this fact, the International Organization for Standardization (ISO) has produced guidelines for good manufacturing practices (ISO 22716:2007) and organic/natural cosmetics (ISO 16128-2:2017), and, despite not having any regulatory power, they have an important role in standardizing cosmetic production [143]. Additionally, in 2007, the International Cooperation on Cosmetics Regulation (ICCR) was established, comprising cosmetic regulatory authorities meeting voluntarily every year to discuss products’ safety and regulations. The conclusions of the meeting are meant to be applied in the members’ legislations [144].

This section aims to summarize some regulatory information about cosmetics and invasive species in some countries: European Union countries, United States of America, Japan and China.

### 5.1. European Union (EU)

Regarding general cosmetic regulations, European Regulation 1223/2009/EC aims to standardize the cosmetic product market to assure human health safety. In the EU, it is mandatory to have a “responsible person” that can also be represented by the importer, distributer or the manufacturer [145]. This identity guarantees that the cosmetic product is submitted through a safety assessment, and, from that, a cosmetic product safety report is produced, which can be found in the product information file (PIF). The PIF should contain all information necessary for the safety assessment and the assessor’s feedback on the product’s safety [143]. The EU regulation also contains annexes with prohibited substances and limited usages of certain colorants, preservatives and UV filters [145]. In the European Union, macroalgae-derived ingredients are accepted in cosmetics and, for example, fucoidan is even present on the EU’s ingredient database, CosIng [146]. The introduction of a cosmetic product in the EU is very influenced by the collection of data and proof to support its claims [145], and, thus, a marine-derived ingredient is obliged to have proven benefits in cosmetics to be introduced in the market.

When it comes to producing a cosmetic using invasive species biomass, European Regulation 1143/2014 states that invasive alien species are not allowed to be sold or bought intentionally, except with a permit. Article 8 states that these permits can be given for research or medicinal purposes, which does not include cosmetics. It is worth mentioning that this Regulation also presents a list of invasive species of Union concern, in which, as of June 2024, contains only two marine species [147]. Furthermore, and specifically in marine habitats, according to Directive 2008/56/EC, EU member states are encouraged to maintain “good environmental status”. This status is defined by various descriptors, one of them being non-indigenous species levels that do not harm ecosystems [148]. Neither of these regulations specify the establishment of a market with the purpose of biomass valorization, and so it would be of great interest to create specific policies for these circumstances [96], as the EU seems compromised with exotic species control.

### 5.2. United States of America (USA)

The American regulations on cosmetics are included in the Federal Food, Drug and Cosmetic Act (FD&C Act), and in December 2022, the Modernization of Cosmetic Regulation Act (MoCRA) was published, tightening the products’ specifications and strengthening Food and Drug Administration (FDA)’s control over cosmetics. This act introduces definitions of possible adverse effects, allowing the FDA to act in case there are serious complications, besides standardizing labels and mandatory registration procedures [149]. However, in the USA, there is no mandatory pre-market approval of ingredients for them to be included in cosmetics, except for color additives. The FDA states that companies are obliged to assure their products’ safety, and measures are only taken if there is evidence that the ingredient is unsafe for human consumption [150], meaning marine-derived ingredients are available in cosmetics.

When it comes to invasive species, the US code Section 666c, titled “Protection of water, oceans, coasts, and wildlife from invasive species”, gives responsibility to the Secretaries of Interior, Army and Agriculture to create projects for invasive species management, taking into consideration the cost-effectiveness of the procedures [151], not mentioning the possibility of marketing byproducts of these species. In fact, each US state has its own further regulations for invasive species. For example, Washington DC classifies the district’s alien species in the categories of Table 3. For levels 1, 2 and 3, these species cannot be possessed or traded without a permit, and only type B can be commercialized, and only if identified with their taxonomic species name [152]. In contrast, Florida’s legislation divides species into prohibited, conditional and non-native, and it is only possible to possess conditional and prohibited species under a permit. In Florida, it is illegal to commercialize any prohibited species, and possession can only be for research, educational exhibition, or eradication and control purposes [153].

Thus, it would be possible to upcycle invasive species biomass in the United States, depending on the state’s classification of the species.

### 5.3. Japan

Cosmetics in Japan have been regulated by the Pharmaceutical and Medical Devices Law since 2014, ensued by the Ministry of Health, Labor, and Welfare, and replacing the previous Pharmaceutical Affair Law [143]. Japan features a distinctive market categorization system, dividing beauty products into cosmetics and quasi-drugs [154]. Similarly to the EU, Japan and China have established prohibited and permitted ingredients in cosmetics, such as preservatives, UV filters and colorants [143].

To register a cosmetic product in Japan, a Cosmetic Manufacturing License and a Cosmetic Marketing License must first be conceded. The Marketing License requires compliance with Good Quality Practice standards, assuring product quality, and the Good Vigilance Practice standard, for post-introduction safety monitoring [143].

Japanese legislation for invasive species is documented in the Invasive Alien Species Act, published in June 2004. In this policy, it is introduced the concept of a permit that allows selling and importation of invasive species. The permit can be conceded for research purposes if it is a part of a mitigation project or if the removal of the species prevents adverse effects in the ecosystem [155].

### 5.4. Republic of China

On 1 January 2021, the new Cosmetic Supervision and Administration Regulation came into effect, representing the profound changes occurring in China’s legislation starting three years prior, in 2018. Following the implementation of this regulation, other subordinate policies were introduced, addressing registration and notification processes, good manufacturing practices, and adverse reaction monitoring, among other areas. As of today, three primary authorities oversee the Chinese cosmetic sector: the State Administration for Market Regulation, the General Administration of Customs and the National Medical Products Administration (NMPA) [143]. In the new 2021 regulation [156], cosmetics can be divided into special and general cosmetics, and all must go through a product safety assessment and submit it after notification or during registration [143].

Regarding Chinese invasive species legislation, in 2022, the central government published the “Measures for the Management of Invasive Species”, ensuing responsibility on county and local governments to act on invasive species. This policy starts by stating that the Ministry of Agriculture and Rural Affairs, together with relevant departments, is responsible for establishing an exotic species database and formulating mitigation and prevention programs. When it comes to marketing these species, there is no specific article mentioning its prohibition, only ‘no unit or individual shall introduce, release or discard alien species without approval’. Finally, it is also given responsibility to the country’s customs, who should act upon illegal introduction, carrying, mailing and smuggling alien species [157]. Since it is a recent law, repurposing invasive species is likely not yet being contemplated in China.

### 5.5. Considerations on Invasive Biomass Valorization and the Role of Policy Makers

Commitment for invasive species control currently represents a global effort, with international conventions, such as the 2022 Convention on Biological Diversity even establishing a 50% reduction in invasive species by 2030. Moreover, marine invasive species also have not gone unnoticed, also being considered in meetings like the Ballast Water Convention and in the Biofouling guidelines [158]. However, by observing the aforementioned legislation, it is clear that actual approved policies in each region are not uniform. Since marine species are much more affected by propagule dispersal, irregular control of their spread turns some region’s efforts fundamentally useless. This phenomenon is also felt throughout the USA, as there are no uniform measures for any invasive species throughout the country [159], and regional managers have no concrete goals or tools to evaluate the effectiveness of their actions [160]. Lastly, the species for which have been control efforts in the EU (*Pterois miles* and *Lagocephalus sceleratus*) are not contemplated in the list, which only brings more inconsistencies within this policy [158]. While it is reasonable to assume that this legislation was only meant to establish the basis for national specific programs, it seems that policy makers did not follow through. Moreover, the preference of flexible and voluntary initiatives for invasive species over binding policies has reportedly shown to lead to inaction and low behavior change [161].

Some proposed changes are fund allocation, improvements in early detection/monitoring/control, improvement of invasion risk assessment and prediction and regulation updates and creation of other policies for invasive species exploitation [158]. It is worth noting that detection, monitoring and early action in invasive species are based on preventive action, taking into consideration that the costs of inaction are much higher than the initial investment.

Thus, these initial procedures must be as reliable as possible, and interdisciplinary research with areas such as biotechnology has a crucial role in the evolution of prediction of invasions and their impacts [4]. With appropriate planning and cost–benefit analysis, it is possible to prioritize research and new projects for invasive species management. This new funding distribution also entails raising the public’s awareness, which is regarded as an extremely important measure by experts [158]. Social and behavioral changes not only encourage debate and change in the governmental authorities [4], but can also present innovative ideas for biomass control, which, in turn, benefits local economies [158].

## 6. Challenges for Algal Biomass Upcycling

Throughout this review, marine-derived ingredients have been explored from the perspective of upcycling invasive species biomass, but there are still barriers to this utilization. Algae have been shown to absorb toxic heavy metals that are abundant in oceans due to high anthropogenic activity [162]. Besides this, high variability between wild specimens could hinder cosmetics’ manufacturing productivity, which is more predictable in laboratory production. When it comes to scaling up to an industrial production, there are other challenges also felt in the production of algal biofuels and feed. Firstly, light and temperature are extremely important factors for algae growth, temperature fluctuations found outdoors being responsible for significant productivity decreases. Secondly, oxygen availability in water needs to be ensured, but not to the point of a high mass transfer, which can cause algal cell damage. The species’ predation and biofouling must also be controlled, which is much more challenging if the biomass is harvested directly from its invasive habitat [163]. In terms of environmental impacts, the addition of fertilizers to increase productivity can risk water quality, and there can be a higher production of greenhouse gases [164]. Overall, the processes of harvesting and cultivation still represent great expenses, mostly due to the specific abiotic factors preferred by each species [164,165]. Even if beach-cast algae were collected, the necessary biomass to create concentrated extracts would be high, since the specimens were not produced in a controlled environment, and, therefore, costs with harvesting and transport would still be significant. These aspects require further research to consider the possibility of creating cosmetics with invasive species’ biomass. Even then, other finalities for this waste are also mentioned throughout the paper.

It is also worth noting the dissonance found in reviewing invasive species databases and literature reviews. This review focused on the most urgent marine invasions to act upon, as their impacts are felt with greater intensity. However, they are not translated into the most investigated or most abundant invasive species, as is evident when comparing Thomsen et al.’s inventory [12] and GISD’s list [21]. Biases should be eliminated in research, as recognized by several authors [13,166], and updated databases should be prioritized.

In addition, it is essential to mention the regulatory simpleness of invasive species and vague measures that are promised throughout legislation. While policy makers recognize the importance of regulating the introduction of exotic species, there is inaction towards the ones already affecting their country or region. Thus, governments must act, creating mitigation programs and allowing for revenue to be generated when controlling invasive species. Considering that eradication takes a lot of resources, this is the ideal opportunity to attempt at implementing a circular economy and, at the same time, managing biodiversity. For example, in the European Union, the legislation does not allow for invasive species biomass upcycling, whether for cosmetics or other uses.

Future perspectives emphasize the need for international collaboration and the development of more effective policies to prevent the spread of invasive species. Advances in technology, such as genetic tools for species identification and modeling techniques for predicting potential invasions, could play a key role in early intervention. Climate change will continue to create new opportunities for invasions, so adaptive management strategies that consider environmental shifts are essential. Public awareness and stakeholder involvement will also be crucial to ensure the success of long-term prevention and mitigation efforts. Thus, while marine invasive species present a persistent threat, the integration of new technologies, adaptive policies, and global cooperation offers hope for more resilient marine ecosystems in the future.

## 7. Conclusions

Invasive species are non-native organisms that, when introduced to a new environment, can harm ecosystems, economies, or human health. This review focused on the invasive species *S. muticum*, *C. taxifolia*, and *U. pinnatifida*. Both *S. muticum* and the green alga *C. taxifolia* demonstrate significant potential as anti-aging and antioxidant ingredients for skincare applications. Similarly, *U. pinnatifida* was highlighted for its high concentrations of antioxidant, skin-brightening and photoprotective compounds. The Azorean archipelago serves as a prime example of the opportunities presented by invasive algal species. Species such as *R. okamurae* and *A. armata* have been identified for their bioactive potential. *R. okamurae*, which has rapidly spread along the coast of São Miguel Island, is rich in polyphenolic compounds with antioxidant properties. Conversely, *A. armata*, now present across all Azorean islands, offers anti-aging and anti-inflammatory benefits. These findings underscore the potential of the Azores for algal biomass upcycling. Not only are these species promising resources, but islands often achieve better outcomes in invasive species management, and there is growing interest among Azorean companies in utilizing algae as a sustainable resource. In conclusion, marine algae represent a valuable opportunity as sustainable cosmetic ingredients, contributing to biodiversity conservation and supporting local economies. However, fostering global collaboration among stakeholders and policymakers is crucial to overcoming the legal and economic challenges.

## Figures and Tables

**Table 2:** Proposed factors for insular susceptibility to invasions and their applications in the Azorean archipelago [109,114].

Islands in General	Azores Islands
Reduced competitive ability	Lack of predators/enemies in Azorean native biota
Relative lack of species diversity	Poor diversity in Azorean native flora and fauna.
Lack of coevolved species	Lack of closely related species in Azorean flora
Relative lack of natural disturbance, such as forest fires	Temperate, humid climate, allowing invasion from species with very different tolerances.
Intensive human disturbance	Exotic species as ornaments in parks and gardens; intense pasture and maize production, hosting several insect pests.

**Table 3:** Washington’s classification of invasive species and their management. Adapted from [152].

Species’ Classification	Invasive Risk	Management
Level 1	High	Prevention and immediate measures
Level 2	High	Long-term disinfestation
Level 3	Moderate to High	Prevention, rapid response or other appropriate measures.
Type A ^1^	Low	Department-approved management of the species’ benefits
Type B	Low or unknown	Can be used in aquariums, live food markets, or as non-domesticated pets.
Type C	Low or unknown	-

^1^ Only for non-native aquatic species that have recognized beneficial properties.

## Data Availability

Not applicable.

## List of Abbreviations

α-MSH	α-melanocyte-stimulating hormone
AP-1	activator protein-1
COX-2	ciclooxigenase-2
DPPH	2,2-diphenyl-1-picrylhydrazyl
ERH	Enemy Release Hypothesis
ERK	extracellular signal-regulated kinase
EU	European Union
FDA	Food and Drug Administration
HAE	hydrothermal extraction
ICCR	International Cooperation on Cosmetics Regulation
IL-1	interleukin 1
ISO	International Organization for Standardization
LPS	lipopolysaccharide
M/G	mannuronic acid and glucuronic acid ratio
MAA	mycosporine-like amino acids
MAPK	mitogen-activated protein kinase
MITF	microphthalmia transcription factor
MMPs	zinc-dependent metalloproteinases
MoCRA	Modernization of Cosmetic Regulation Act
MUFA	monounsaturated fatty acid
NF-κB	nuclear factor kappa B
NMPA	National Medical Products Administration
Nrf2	nuclear factor erythroid 2-like
NWH	Novel Weapons Hypothesis
PIF	product information file
PUFA	polyunsaturated fatty acids
ROS	reactive oxygen species
SAE	supercritical carbon dioxide extraction
SIRT1	sirtuin 1
TNF-α	tumor necrosis factor-alpha
TPM	tetraprenyltoluquinol chromane meroterpenoid
TRP	tyrosinase related proteins
USA	United States of America
UV	ultraviolet
